# Pediatric severe sepsis: epidemiology and risk factors associated with acute kidney injury

**DOI:** 10.3389/fped.2025.1736473

**Published:** 2026-01-21

**Authors:** Haibo Li, Ying Zhang, Hongyan Zhu, Ran Yu, Qi Zhou, Jiannan Song, Jiannan Wu, Wanli Ma, Zhanfei Hu, Jian Wang, Xuegao Yu, Hongyu Zhang

**Affiliations:** 1 Department of Anesthesiology, Affiliated Chifeng Clinical Medical College of Inner Mongolia Medical University, Chifeng Municipal Hospital, Chifeng, China; 2 Department of Anesthesiology, Affiliated Chifeng Clinical Medical College of Inner Mongolia Medical University, Chifeng, China; 3 Division of Orthopaedic Surgery, Department of Orthopaedics, Nanfang Hospital, Southern Medical University, Guangzhou, Guangdong, China; 4 Department of Laboratory Medicine, The First Affiliated Hospital, Sun Yat-sen University, Guangzhou, Guangdong, China; 5 Five Senses Ward, Nanfang Hospital Zengcheng Campus, Southern Medical University, Guangzhou, Guangdong, China

**Keywords:** acute kidney injury, child, mortality, risk factors, severe sepsis

## Abstract

**Background:**

Pediatric severe sepsis (PSS) is one of the leading causes of morbidity and mortality in children, incurring substantial social costs. Acute kidney injury (AKI) plays a critical role in determining PSS severity and prognosis. However, existing literature provides limited data regarding the risk factors associated with AKI in pediatric sepsis patients and the impact of AKI on hospital outcomes for these patients. This study aimed to analyze the temporal trends in incidence and outcomes of AKI among hospitalized PSS patients from 2010 to 2019, and identify associated risk factors; and assess the impact of AKI on in-hospital mortality and healthcare resource utilization.

**Methods:**

This study utilized the nationally representative National Inpatient Sample (NIS) database of the United States to conduct a retrospective analysis. All children aged 0 (infants) to 18 years who were diagnosed with severe sepsis between 2010 and 2019 were included. Patients were grouped by AKI status, and in-hospital mortality and medical resource utilization (length of stay and inflation-adjusted costs) were compared. Multivariate regression identified AKI risk factors.

**Results:**

The incidence rate of AKI among hospitalized PSS patients increased from 2.7% in 2010 to 8.0% in 2019. However, in-hospital mortality declined from 32.40% to 17.90% over the same period. The incidence of AKI was significantly higher in patients with comorbidities. Studies have shown that hospitalizations associated with AKI have a higher likelihood of involving infection sites and a variety of pathogenic flora.

**Conclusion:**

While the incidence of AKI increased from 2010 to 2019, associated mortality decreased. This likely reflects advancements in critical care that are improving survival, even as more cases are recognized. AKI, affecting 5% of PSS patients, remained a potent marker of severity, was associated with a sevenfold increased risk of mortality and driven by identifiable risk factors like specific comorbidities and infections. Enhanced early identification of at-risk children is crucial to further improve outcomes.

## Introduction

Sepsis is a condition characterized by a malfunctioning immune reaction to infection, the dysregulation between the pro—coagulant and anticoagulant systems within the body is acknowledged as one of the pathogenic reasons underlying sepsis, resulting in potentially fatal organ dysfunction (1, 2). And the global health equity gap is particularly detrimental to children, increasing their susceptibility to infectious diseases. Studies have pointed out that each year, 50 million people worldwide are infected with sepsis, of whom half are newborns and children under the age of 19 (3). Pediatric severe sepsis (PSS) is a life-threatening condition and one of the leading causes of mortality in children worldwide. The prevalence of severe sepsis has been reported to be increasing in recent years (4), due to rising complications (5, 6), more frequent multidrug—resistant and opportunistic infections (7, 8), and improved surveillance and diagnosis (9, 10). Among the series of pathological changes caused by severe sepsis, the kidneys are among the most frequently affected organs. However, the diagnosis of AKI relies on changes in serum creatinine and urine output—indicators that are often lagging and non-specific, making early recognition and timely intervention particularly challenging (11, 12). In addition to insufficient renal perfusion, microvascular dysfunction, inflammation, and the response of cells to inflammatory injury can all contribute to sepsis-related acute kidney injury, which may progress to chronic kidney disease, imposing a substantial long-term health burden—a sequela often more significant than that of resolved dysfunction in other organ systems (13, 14). Children suffering from this severe illness generally face a heightened risk of sepsis-associated acute kidney injury (SA—AKI) (15, 16), which further elevates morbidity and mortality (17). Nevertheless, data on AKI's impact on hospital outcomes in PSS inpatients and its associated factors remain scarce.

The primary objectives of our study were as follows: (1) to evaluate the temporal trends in incidence and outcomes of PSS patients with concurrent AKI, as well as the associated factors; and (2) to determine the impact of AKI on sepsis patients during the same time period.

## Methods

### Study plan and source of data

The data was sourced from the National Inpatient Sample (NIS), which is part of the Healthcare Cost and Utilization Project. The NIS contains patient demographics, medical conditions, treatments, duration of stay, costs, insurance types, and mortality information. We conducted a retrospective analysis using a large sample spanning 2010–2019. As the study involved secondary analysis of de-identified data, institutional review board approval was not required. All patient data were anonymized, waiving the need for informed consent.

### Study population

**Figure 1:**
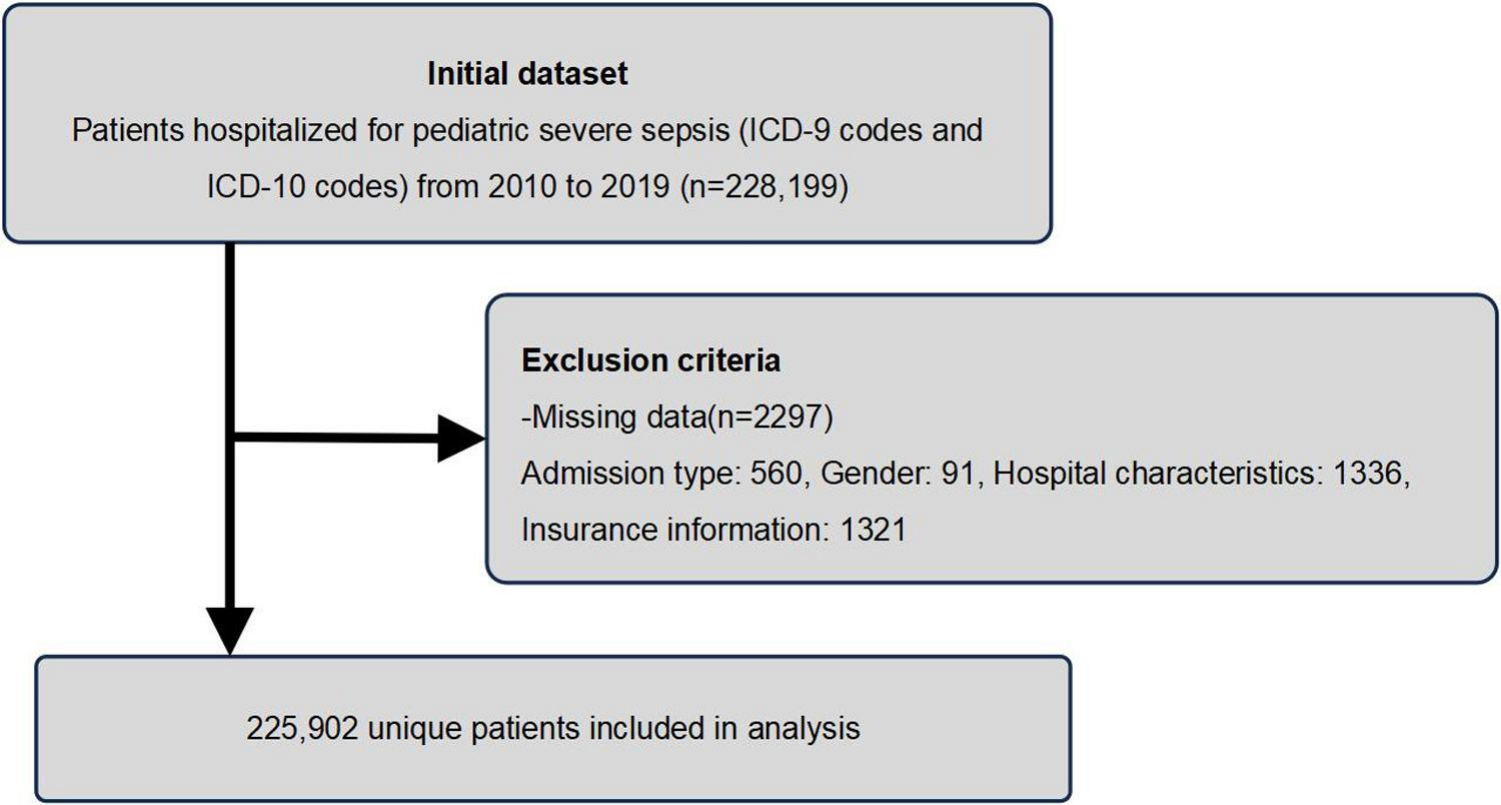
Flow chart of the patient selection process in this study.

The study included pediatric patients (0–18 years) hospitalized for sepsis between 2010 and 2019. To avoid potential bias from repeated measures, only the first recorded hospitalization for sepsis within the study period was included for each unique patient. To examine demographic features, we excluded patients with missing information on sex, insurance type, elective admission, and hospital profile (including patients with duplicate missing data). Given that there are many patients lacking racial data, we were classify these patients into the “Other” category to preserve sample size. After exclusions, 225,902 PSS inpatients were included (Figure 1). For the purpose of this analysis, patients under 1 year of age were categorized in the “<1 year” group. Since this study did not assess AKI severity and in consideration of the administrative nature of the NIS database, AKI and sepsis was defined by the presence of any relevant diagnostic code (ICD-9-CM and ICD-10-CM) in the primary or secondary position during the hospitalization (Supplementary Tables 1–3).

### Outcome measures

The primary objective was to analyze epidemiological characteristics of PSS patients with and without AKI from 2010 to 2019, focusing on hospitalization status and mortality. In-hospital mortality was the primary outcome. Secondary outcomes were medical resource utilization, assessed by median length of stay and inflation-adjusted hospitalization costs. Comorbidities and infection-related data (site and pathogen) were also analyzed.

### Statistical analysis

Categorical variables will be described as proportions and percentages, while continuous variables will be presented as medians and interquartile ranges. The *χ*^2^ test compared categorical variables; the Mann–Whitney *U* test compared quantitative data. Given the large sample size of this study (*n* = 225,902), to identify the most robust and clinically relevant predictors for the multivariate model and to mitigate the risk of model overfitting, we employed a more stringent entry criterion of *p* ≤ 0.001 from univariate analyses. Variables meeting this threshold were subsequently entered into a multivariate model, which will be adjusted for age, sex, race, median household income, payer status, type of admission, hospital characteristics including bed size, location/teaching status, and regions, and all baseline comorbidities. This adjustment is intended to significantly reduce the impact of other confounding factors.

In addition, annual percentage changes in PSS morbidity and mortality (with/without AKI) were calculated. The distributions of length of stay and hospitalization costs were positively skewed and contained extreme outliers—typical for healthcare utilization data—we therefore used medians to analyze trends, which are resistant to the influence of extreme values. We used time series analysis for evaluating trends of incidence rate, length of stay, and hospitalization charges during the study period.

## Results

### Baseline characteristics

Table 1 summarizes the basic characteristics of these patients. The overall incidence of acute kidney injury (AKI) in PSS patients was 4.8% (*n* = 10,952). Of 225,902 sepsis patients, 171,622 (75.97%) were infants <1 year. A higher proportion of PSS—AKI patients were male (53.64% vs. 46.36%, *P* < 0.001) and White (40.38%, *P* < 0.001). In addition, the median age of admission for PSS—AKI patients was 3 years (IQR: 0–17 years, *P* < 0.001), and most patients were covered by Medicaid (57.39%). At the same time, PSS—AKI patients were mainly admitted to urban medical institutions (99.22%) and were mostly treated in hospitals with larger beds (64.05%).

**Table 1:** Baseline characteristics of sepsis hospitalizations with and without acute kidney injury (2010-2019).

Characteristics	AKI	No AKI	*P*
Total (*n* = 225, 902)	10952	214,950	
Total incidence (%)	4.8		
Age (year) at admission Median (IQR)	3 (0–17)	0 (0–13)	<0.001
Age group (%)			<0.001
<1 year	39.69	77.82	
1–3	11.47	7.83	
4–6	7.21	3.26	
7–10	9.75	3.60	
11–18	31.88	7.48	
Gender (%)			<0.001
Male	53.64	55.23	
Female	46.36	44.77	
Race (%)			<0.001
White	40.38	41.36	
Black	19.46	17.11	
Hispanic	20.90	21.99	
Asian or Pacific Islander	3.44	3.97	
Native American	0.99	0.88	
Other	14.84	14.69	
Type of insure (%)			<0.001
Medicare	0.72	0.32	
Medicaid	57.39	56.38	
Private insurance	34.98	35.84	
Self-pay	1.92	3.13	
No charge	0.05	0.12	
Other	4.95	3.76	
Bed size of hospital (%)			<0.001
Small	14.56	13.74	
Medium	21.38	26.81	
Large	64.05	59.46	
Elective admission (%)	6.59	3.46	<0.001
Type of hospital (teaching %)	95.01	72.50	<0.001
Location of hospital			<0.001
Urban (%)	99.22	95.25	
Rural (%)	0.78	4.75	
Region of hospital (%)			<0.001
Northeast	13.74	15.06	
Midwest or North Central	22.81	18.15	
South	40.54	42.46	
West	22.91	24.32	
Outcome			
Died (%)	21.89	3.07	<0.001
LOS, median (IQR), d	17 (1–145)	7 (1–85)	<0.001
TOTCHE, median (IQR)	$235,912 ($14,327–2,383,936)	$43,077 ($4,513–755,247)	<0.001

LOS, length of stay; TOTCHE, total charge; IQR, interquartile range. *P* < 0.001 indicates statistical significance.

### Trend analysis

**Figure 2:**
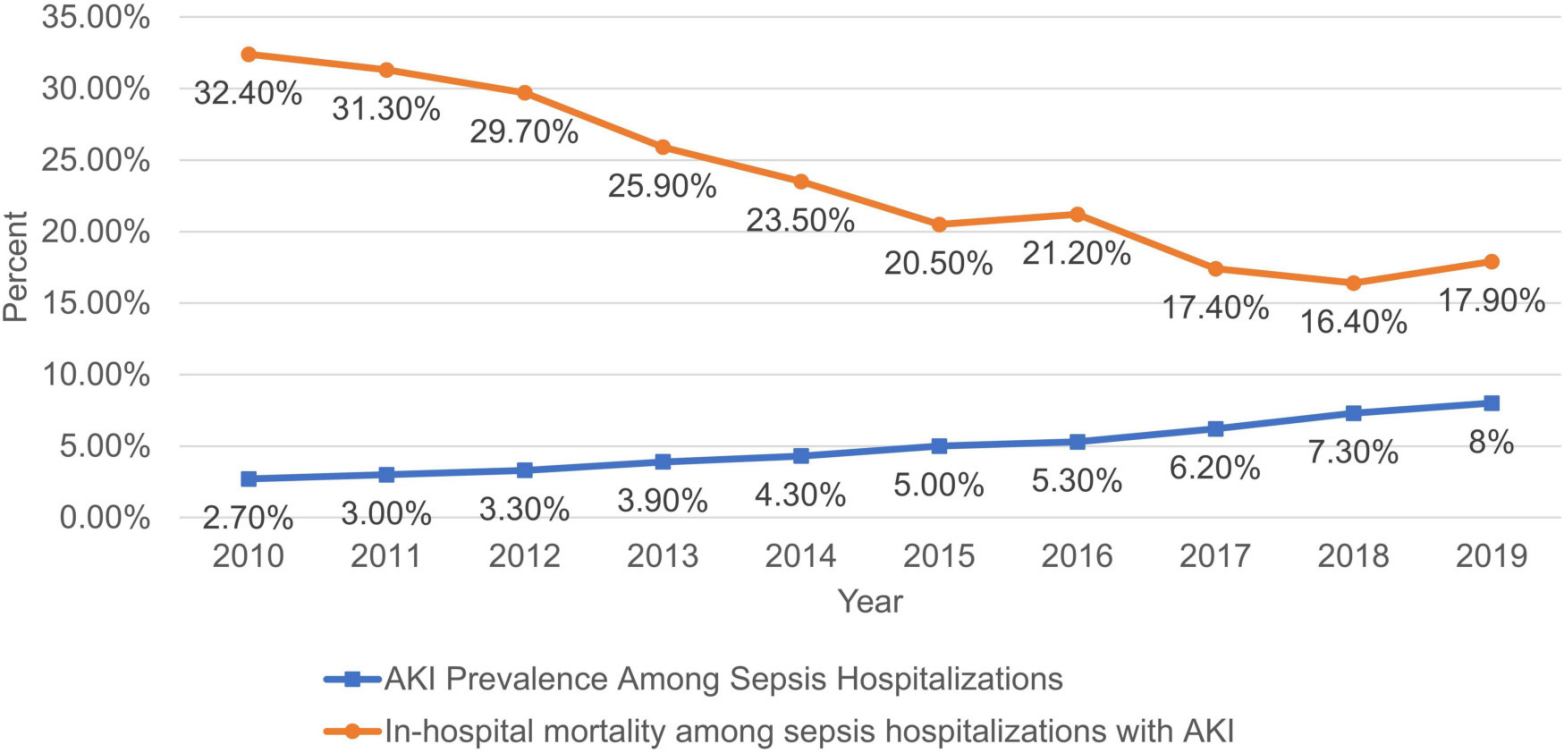
Incidence and mortality of AKI in hospitalized patients with PSS from 2010 to 2019.

**Figure 3:**
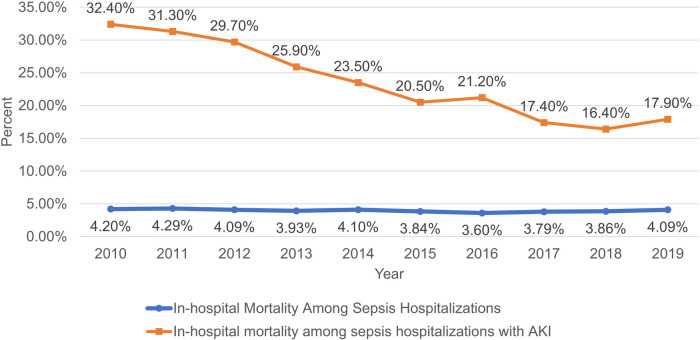
Annual mortality with and without AKI in hospitalized patients with PSS.

**Figure 4:**
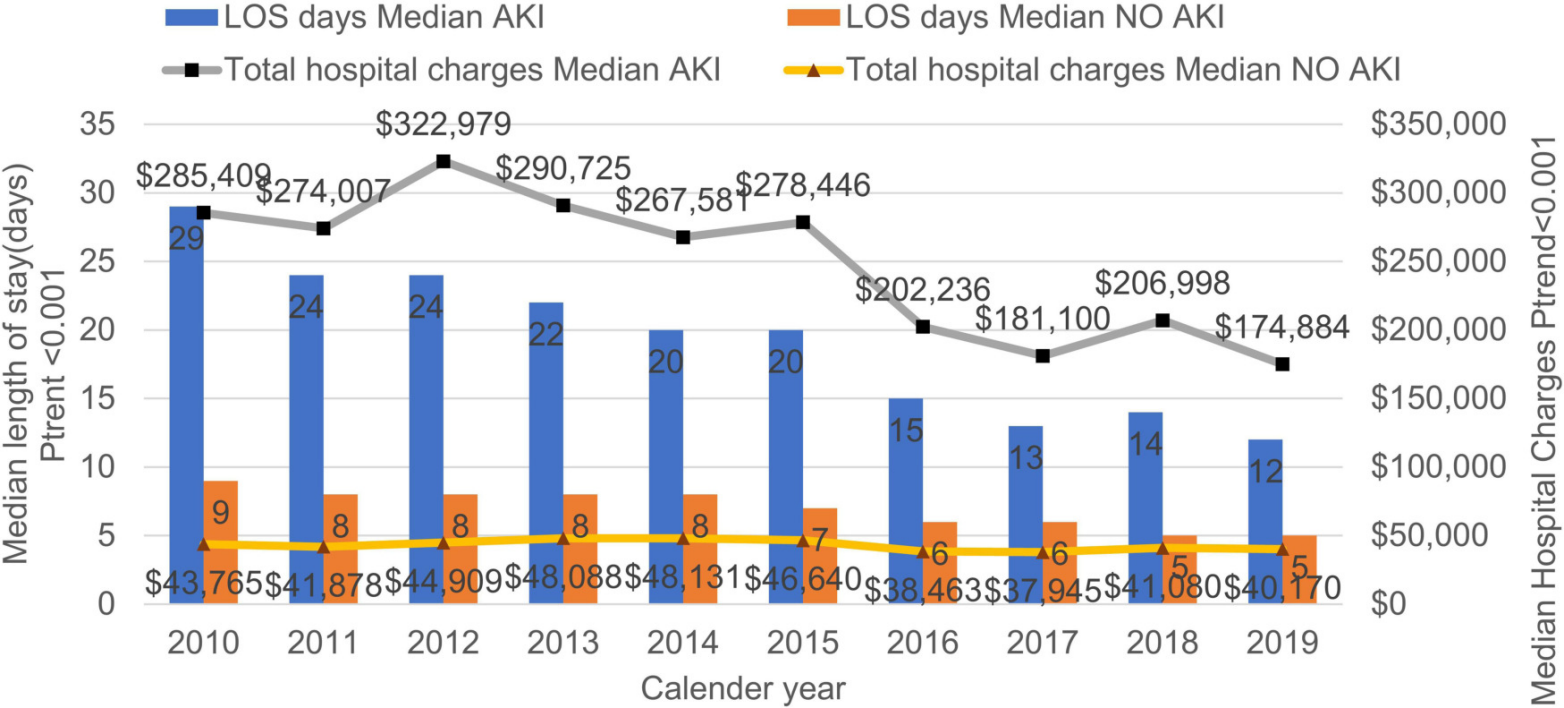
The median length of hospital stay and the median total hospital costs of hospitalized PSS patients with and without AKI from 2010 to 2019.

The incidence of AKI among PSS in—patients has demonstrated a remarkable upward tendency. It rose from 2.7% in 2010 to 8% in 2019 (*P* < 0.001). Conversely, mortality declined from 32.4% to 17.9% (*P* < 0.001) (Figure 2). Annual in-hospital mortality was lower in PSS patients without AKI (3.07% vs. 21.89%, *P* < 0.001), though both groups showed decreasing mortality over time (Figure 3). Supplementary Table 4 shows the demographic data of PSS—AKI patients from 2010 to 2019. The total number of pediatric sepsis-related AKI hospitalizations gradually increased. As shown in Table 1 and Figure 4, PSS—AKI patients had significantly longer median hospital stays (17 vs. 7 days) and higher median costs ($235,912 vs. $43,077) (*P* < 0.001).

### Comorbidities

The research findings indicate that PSS—AKI patients had a significantly higher associations of developing hypertension, encompassing both simple and complex types (13.51% vs. 1.76%), congestive heart failure (5.98% vs. 0.56%), rheumatoid arthritis/collagen vascular diseases (0.73% vs. 0.10%), peripheral vascular diseases (2.78% vs. 0.41%), and valvular diseases (6.92% vs. 1.76%) (*P* < 0.001) (Table 2).

**Table 2:** Comorbidities in sepsis hospitalizations with and without Acute kidney injury.

Comorbidities	AKI	No AKI	*P*
Cardiovascular			
Hypertension	13.51	1.76	<0.001
Congestive heart failure	5.98	0.56	<0.001
Rheumatoid arthritis/collagen vascular diseases	0.73	0.10	<0.001
Peripheral vascular disorders	2.78	0.41	<0.001
Valvular disease	6.92	1.76	<0.001
Pulmonological			
Pulmonary circulation disorders	4.52	1.11	<0.001
Chronic pulmonary disease	7.52	3.06	<0.001
Psychiatric			
Depression	2.31	0.44	<0.001
Alcohol abuse	0.09	0.01	<0.001
Drug abuse	2.95	0.54	<0.001
Paralysis	6.66	1.78	<0.001
Psychoses	0.96	0.18	<0.001
Endocrinological			
Acquired immune deficiency syndrome	0.03	0.01	0.152
Diabetes, uncomplicated	1.61	0.30	<0.001
Diabetes with chronic complications	0.76	0.22	<0.001
Hypothyroidism	3.38	0.99	<0.001
Obesity	3.75	0.67	<0.001
Weight loss	8.83	1.88	<0.001
Neurologic			
Other neurological disorders	15.93	3.94	<0.001
Renal			
Renal failure	6.44	0.60	<0.001
Gastrointestinal			
Peptic ulcer disease excluding bleeding	0.13	0.03	<0.001
Liver disease	6.58	0.98	<0.001
Blood			
Deficiency anemias	8.95	2.69	<0.001
Chronic blood loss anemia	0.85	0.24	<0.001
Coagulopathy	33.43	4.99	<0.001
Fluid and electrolyte disorders	65.63	17.67	<0.001

*P* < 0.001 indicates statistical significance.

Compared with non—AKI patients hospitalized for sepsis, AKI patients may also have potential associations with the following comorbidities: pulmonary circulatory disorders (4.52% vs. 1.11%), chronic lung diseases (7.52% vs. 3.06%), uncomplicated diabetes (1.61% vs. 0.30%), chronic complications of diabetes (0.76% vs. 0.22%), hypothyroidism (3.38% vs. 0.99%), other neurological disorders (15.93% vs. 3.94%), digestive ulcers without bleeding (0.13% vs. 0.03%), liver diseases (6.58% vs. 0.98%), anemia deficiency (8.95% vs. 2.69%), chronic hemorrhagic anemia (0.85% vs. 0.24%), and electrolyte disturbances (65.63% vs. 17.67%), and the like (Table 2).

As presented in Table 3, the correlation between AKI and comorbid conditions spanning from 2010 to 2019 is depicted. The odds ratio (OR) associated with the comorbidity of fluid and electrolyte imbalances within the PSS—AKI group was the greatest, followed by rheumatoid arthritis/collagen vascular diseases (OR 2.970; 95% CI 1.88–4.68) and coagulation disorders (OR 2.846; 95% CI 2.59–3.12) (all *P* < 0.001). Other common comorbidities included uncomplicated diabetes (OR, 2.704; 95% CI, 2.09–3.51), cardiac arrest (OR, 2.445; 95% CI, 2.01–2.97), obesity (OR, 2.330; 95% CI, 1.97–2.76), congestive heart failure (OR, 2.169; 95% CI, 1.46–3.23) (*P* < 0.001), etc. (Table 3).

**Table 3:** Relationship between acute kidney injury and comorbidities.

Comorbidities	Multivariate logistic regression		
	OR	95% CI	*P*
Cardiovascular			
Hypertension	1.750	1.57–1.95	<0.001
Congestive heart failure	2.169	1.46–3.23	<0.001
Acute myocardial infarction	3.272	0.98–10.93	0.054
Rheumatoid arthritis/collagen vascular diseases	2.970	1.88–4.68	<0.001
Peripheral vascular disorders	1.191	0.91–1.56	0.205
Deep vein thrombosis	1.891	1.57–2.27	<0.001
Valvular disease	1.864	1.65–2.10	<0.001
Peripheral vascular disease	1.313	1.01–1.71	0.044
Cardiac arrest	2.445	2.01–2.97	<0.001
Arrhythmia	1.165	0.67–2.02	0.585
Heart failure	1.249	0.81–1.92	0.310
Pulmonological			
Pulmonary circulation disorders	0.968	0.81–1.16	0.727
Respiratory disease	1.572	1.27–1.95	<0.001
Pneumonia	1.281	1.18–1.40	<0.001
Chest pain	2.221	1.33–3.72	0.002
Respiratory failure	1.038	0.93–1.16	0.521
Continuous trauma ventilation	0.781	0.71–0.86	<0.001
Chronic pulmonary disease	1.093	0.98–1.22	0.117
Pulmonary embolism	1.119	0.77–1.63	0.555
Psychiatric			
Depression	1.683	1.35–2.10	<0.001
Alcohol abuse	3.816	0.97–15.15	0.057
Drug abuse	1.254	1.02–1.54	0.030
Paralysis	1.204	1.06–1.37	0.005
Psychoses	1.678	1.11–2.53	0.013
Endocrinological			
Acquired immune deficiency syndrome	0.543	0.11–2.71	0.457
Diabetes, uncomplicated	2.704	2.09–3.51	<0.001
Diabetes with chronic complications	1.000	0.72–1.39	0.999
Hypothyroidism	1.188	0.99–1.42	0.060
Obesity	2.330	1.97–2.76	<0.001
Weight loss	1.301	1.17–1.45	<0.001
Neurologic			
Acute cerebrovascular disease	1.375	1.08–1.75	0.010
Other neurological disorders	1.863	1.70–2.05	<0.001
Renal			
Renal failure	4.331	3.71–5.06	<0.001
Gastrointestinal			
Gestrointestinal complication	0.886	0.74–1.07	0.202
Peptic ulcer disease excluding bleeding	1.296	0.60–2.82	0.514
Gastrointestinal bleeding	1.916	1.41–2.60	<0.001
Liver disease	1.904	1.67–2.17	<0.001
Blood			
Deficiency anemias	1.178	1.00–1.39	0.058
Chronic blood loss anemia	1.198	0.81–1.77	0.364
Blood transfusion	1.707	1.58–1.85	<0.001
Coagulopathy	2.846	2.59–3.12	<0.001
Thrombocytopenia	0.896	0.79–1.01	0.076
Fluid and electrolyte disorders	4.045	3.80–4.31	<0.001
Tumor			
Lymphoma	0.576	0.39–0.84	0.005
Metastatic cancer	1.023	0.74–1.42	0.890
Solid tumor without metastasis	0.628	0.50–0.79	<0.001

OR, odds ratio; CI, confidence interval. Multivariate logistic regression model was adjusted for age, sex, race, median household income, payer status, type of admission, hospital characteristics including bed size, location/teaching status, and regions, and all baseline comorbidities. *P* < 0.001 indicates statistical significance.

### Infections associated with PSS—AKI hospitalizations

Regarding infection sites (Table 4), gastrointestinal infections (13.28% vs. 9.47%) and endocarditis (0.66% vs. 0.08%) were more common in PSS-AKI hospitalizations (*P* < 0.001). Among pathogens, Staphylococcus aureus (7.13% vs. 2.81%), Escherichia coli (6.32% vs. 3.13%), Streptococcus (4.47% vs. 2.24%), Candida (1.70% vs. 0.36%), Pseudomonas (1.65% vs. 0.36%), Enterococcus (1.48% vs. 0.46%), anaerobic bacteria (0.40% vs. 0.14%), and Salmonella (0.35% vs. 0.11%) were relatively common (*P* < 0.001). The results of Table 5 indicate that, in the correlation analysis of PSS complicated by AKI and the sites of infection, only the factor of urinary tract infection exhibits statistical significance (OR = 1.408; 95% CI: 1.30–1.53; *P* < 0.001). For pathogens, Escherichia coli (*E. coli*) (OR = 2.003; 95% CI: 1.77–2.27; *P* < 0.001) and Staphylococcus aureus (OR = 1.387; 95% CI: 1.23–1.56; *P* < 0.001) were the most predominant pathogenic bacteria.

**Table 4:** Infection in sepsis hospitalizations with and without acute kidney injury.

Variables	AKI	No AKI	*P*
Infection sites (%)			
Gastrointestinal infection	13.28	9.47	<0.001
Endocarditis infection	0.66	0.08	<0.001
Skin tissue infection	0.16	0.13	0.406
Joint bone tissue infection	0.02	0.01	0.163
Intracranial infection	0.11	0.04	0.003
Bacterial infection (%)			
*Staphylococcus aureus*	7.13	2.81	<0.001
*Escherichia coli [E. coli]*	6.32	3.13	<0.001
*Streptococcal*	4.47	2.24	<0.001
*Candidal*	1.70	0.36	<0.001
*Pseudomonas*	1.65	0.36	<0.001
*Enterococcus*	1.48	0.46	<0.001
*Anaerobes*	0.40	0.14	<0.001
*Salmonella*	0.35	0.11	<0.001
*Actinomycotic*	0.03	0.004	0.038
*Gonococcal*	0.00	0.01	1.000

*P* < 0.001 indicates statistical significance.

**Table 5:** Relationship between acute kidney injury and infection.

Variable	Multivariate logistic regression		
	OR	95% CI	*P*
Infection sites			
Urinary tract infection	1.408	1.30–1.53	<0.001
Wound infection	1.044	0.87–1.25	0.638
Lung infection	1.538	0.17–13.72	0.700
Gastrointestinal infection	0.972	0.91–1.04	0.412
Skin tissue infection	0.952	0.56–1.61	0.856
Joint bone tissue infection	0.923	0.18–4.73	0.924
Endocarditis infection	0.821	0.57–1.18	0.283
Intracranial infection	0.776	0.39–1.55	0.472
Bacterial infection			
*Enterococcus*	0.977	0.75–1.27	0.865
*Streptococcal*	1.277	1.10–1.48	0.001
*Salmonella*	0.997	0.58–1.70	0.990
*Pseudomonas*	1.349	1.05–1.74	0.021
*Escherichia coli [E. coli]*	2.003	1.77–2.27	<0.001
*Anaerobes*	1.054	0.63–1.76	0.839
*Staphylococcus aureus*	1.387	1.23–1.56	<0.001
*Candidal*	1.154	0.89–1.50	0.283

OR, odds ratio; CI, confidence interval. Multivariate logistic regression model was adjusted for age, sex, race, median household income, payer status, type of admission, hospital characteristics including bed size, location/teaching status, and regions, and all baseline comorbidities. *P* < 0.001 indicates statistical significance.

### Multivariate predictors of in-hospital mortality in PSS—AKI patients

Table 6 compares predictors of mortality in PSS—AKI patients. The highest in-hospital mortality rate occurred in patients aged 11–18 years (OR, 4.317; 95% CI: 4.05–4.61; *P* < 0.001), and among black individuals (OR, 1.13; 95% CI: 1.06–1.20; *P* < 0.001). In addition, multiple comorbidities significantly increased the risk of mortality during hospitalization for patients with sepsis and acute kidney injury.

**Table 6:** Multivariable predictors of in-hospital mortality in sepsis hospitalizations with acute kidney injury.

Variable	Multivariate logistic regression		
	OR	95% CI	*P*
Age			
<1 year	Ref	–	–
1–3	1.394	1.29–1.50	<0.001
4–6	2.205	2.01–2.42	<0.001
7–10	2.616	2.40–2.85	<0.001
11–18	4.317	4.05–4.61	<0.001
Female	0.993	0.95–1.04	0.758
Race			
White	Ref	–	–
Black	1.13	1.06–1.20	<0.001
Hispanic	0.908	0.86–0.96	0.002
Asian or Pacific Islander	0.928	0.82–1.05	0.226
Native American	1.100	0.88–1.37	0.401
Other	1.017	0.95–1.09	0.611
Number of Comorbidity			
0	Ref	–	–
1	2.471	2.29–2.67	<0.001
≥2	3.120	2.80–3.48	<0.001
Type of insurance			
Medicare	Ref	–	–
Medicaid	1.903	1.42–2.56	<0.001
Private insurance	1.798	1.34–2.42	<0.001
Self-pay	1.463	1.05–2.04	0.025
No charge	0.546	0.21–1.45	0.226
Other	2.014	1.48–2.75	<0.001
Bed size of hospital			
Small	Ref	–	–
Medium	0.800	0.74–0.86	<0.001
Large	1.047	0.98–1.12	0.156
Elective admission	0.833	0.76–0.91	<0.001
Teaching hospital	3.015	2.73–3.33	<0.001
Urban hospital	1.269	1.00–1.61	0.051
Region of hospital			
Northeast	Ref	–	–
Midwest or North Central	1.226	1.14–1.32	<0.001
South	1.169	1.09–1.25	<0.001
West	1.181	1.10–1.27	<0.001

OR, odds ratio; CI, confidence interval.

## Discussion

Epidemiological characteristics of pediatric severe sepsis vary across studies, likely due to differences in populations, observation periods, and diagnostic criteria. We analyzed 225,902 associated hospitalizations of patients with sepsis aged 0–18 years between 2010 and 2019. Key findings included: (1) The incidence of AKI in PSS patients increased nearly three times (from 2.7% to 8.0%), while in-hospital mortality decreased by nearly half (from 32.4% to 17.9%); (2) PSS—AKI patients had significantly longer hospital stays (17 vs. 7 days) and higher hospitalization costs ($235,912 vs. $43,077) than non—AKI patients, though both metrics showed a downward trend over time; (3) AKI risk was strongly associated with specific comorbidities (e.g., fluid-electrolyte disturbances, coagulation disorders), infection sites (e.g., gastrointestinal infections), and pathogens (e.g., Escherichia coli, Staphylococcus aureus); (4) We observed that among patients with PSS—AKI, the most common age groups were infants <1 year (39.69%) and 11–18 years (31.88%), and the incidence rate was higher in male patients (53.64% vs. 46.36%); (5) Those with multiple comorbidities had the highest in-hospital mortality.

Our study highlights a rising incidence of AKI in PSS inpatients over a decade, alongside declining mortality—a trend consistent with other studies, including in adults (4, 18, 19). This trend may reflect increased global attention to pediatric sepsis, promoting earlier and more aggressive management. Advances in diagnosis, monitoring, and treatment may also have mitigated disease severity. Thus, while overall AKI incidence increased, mortality declined. We also found that hospital stays and costs were substantially higher for PSS—AKI patients, though both decreased over time. Optimizing management to prevent AKI progression may help reduce length of stay and costs.

In the European Children's Infectious Disease Study (EUCLIDS), half of the children were confirmed to have invasive bacterial infections, and one-third of the survivors were discharged with disabilities (20). Numerous studies have shown that early identification of pathogens and timely administration of antibiotics are crucial and have beneficial effects on prognosis (21–23). In our study, Staphylococcus aureus, Escherichia coli and Streptococcus were prevalent; however, it should be noted that these pathogen-specific findings are based on clinical diagnosis codes rather than laboratory-confirmed microbiology. While this may not fully capture the true etiologies, the patterns observed can still offer valuable references for clinical prevention and intervention. Among all infection sites, gastrointestinal infections showed the closest association with PSS—AKI. It has been reported that factors such as a younger age, blood—related or immunological comorbidities, malignancies, and abdominal infections are linked to severe AKI (24), and in our study we noted an increased proportion of AKI in patients with water and electrolyte disorders, coagulation disorders, other neurological disorders, hypertension, chronic lung disease, valvular disease, paralysis, congestive heart failure. Among them, fluid and electrolyte imbalance, which is a recognized risk factor, usually reflects the cumulative effects of capillary leakage, active resuscitation and potential disease severity in sepsis. Coagulation dysfunction is also prominent in our model. It is a known component of organ dysfunction caused by sepsis and can exacerbate renal microvascular damage (25, 26). Reducing these risk factors and implementing early interventions may slow PSS—AKI progression.

In summary, integrating these data-driven strategies into clinical practice is crucial. This entails enhanced monitoring of high-risk patients—clinicians should maintain a heightened suspicion for AKI in neonates, male patients, and those with specific comorbidities such as fluid-electrolyte imbalances, coagulation disorders, or hypertension. Concurrently, focused attention on high-risk pathogens and infection sites necessitates proactive preventive measures and the prompt administration of appropriate antibiotic therapy. Implementing such a targeted approach may help mitigate the progression of PSS—AKI, thereby potentially alleviating its substantial healthcare burden and reducing associated mortality.

We utilized a large, nationally representative database to accurately capture real-world trends in morbidity and mortality. However, this study has several potential limitations. First, the retrospective, administrative nature of the data introduces the possibility of recording bias due to inconsistent identification and coding practices, which may lead to an underestimation of AKI incidence. Moreover, the database contains only in-hospital information, lacking post-discharge follow-up and out-of-hospital mortality data, thus providing an incomplete clinical picture. Second, we lacked data on specific exposures such as nephrotoxic medications and radiocontrast agents, which are known risk factors for AKI. Third, important treatment variables that influence sepsis outcome—including mechanical ventilation and renal replacement therapy—were not available for adjustment in our multivariate models, potentially confounding the observed association between AKI and mortality. Fourth, we could not fully address the interplay between comorbidities and pre-existing chronic kidney disease (CKD); excluding patients with pre-existing CKD or a history of AKI can mitigate such bias and enhance the validity of the findings. Fifth, although immunocompromised children were included in the cohort, we did not perform a dedicated subgroup analysis for this population, so our results may not fully reflect their distinct risk profile and outcomes. An additional limitation is the granularity of age data within this group; the use of a single “<1 year” category, necessitated by the database structure, precludes a more nuanced analysis distinguishing neonates from older infants. What's more, the risk factors, prevalence, clinical and microbiological etiology of newborns and infants (<1 year old) are significantly different from those of older children and adolescents, and their pathophysiological mechanisms are also quite distinct (27, 28), but we do not exclude them.

## Conclusion

Between 2010 and 2019, the incidence of PSS with or without AKI increased among hospitalized children. AKI was associated with higher mortality, morbidity, longer hospital stays, and greater resource use, increasing public health burden. However, with the deepening of pediatric sepsis research and the improvement of medical level, the overall mortality rate of PSS has decreased, especially the mortality rate of PSS patients with AKI has shown a significant decreasing trend. Therefore further research and technological innovation are necessary to improve early diagnosis of AKI, identification of risk factors, and timely implementation of preventive treatment, This is of crucial significance for improving children's health and reducing the risk of adverse consequences caused by infections and related diseases.

## Data Availability

The raw data supporting the conclusions of this article will be made available by the authors, without undue reservation.
