# Supplementary material for: Pediatric severe sepsis: epidemiology and risk factors associated with acute kidney injury
Source: Front Pediatr. 2026 Jan 21;13:1736473. doi: 10.3389/fped.2025.1736473 (PMC12868292; doi:10.3389/fped.2025.1736473)
Supplement: Supplementary file 1 [file Datasheet1.pdf]

Supplementary Table 1. ICD-9-CM and ICD-10-CM Codes of sepsis and AKI

Variable	ICD-9-CM Codes	ICD-10-CM Codes
Sepsis	67020, 67022, 67024, 77181, 99591, 99592,0031, 0223, 0270, 0271, 0380, 03810, 03811, 03812, 03819, 0382, 0383, 03840, 03841, 03842,03843, 03844, 03849, 0388, 0389, 09889, 1125, 78552, 0270, 0271, 09889	O85, P360, P3610, P3619, P362, P3630, A084, A088, T86852, A08, A04, A044, A049, A048, P3639, P364, P365, P368, P369, A021, A227, A267, A327, A400, A401, A403, A408, A409, A4101, A4102, A411, A412, A413, A414, A4150, A4151, A4152, A4153, A4159, A4181, A4189, A419, A427, A5486, B377, R6520, R6521, A021, A320, A3211, A3212, A3281, A3282, A3289, A329, A260, A268, A269, A5482, A5484, A5489, A549
AKI	5845, 5846, 5847, 5848, 5849, 5836, 5837	N170, N171, N172, N178, N179

AKI: Acute kidney injury

ICD-9-CM, International Classification of Diseases 9th revision Clinical Modification;

ICD-10-CM, International Classification of Diseases 10th revision Clinical

Supplementary Table 2. ICD-10-CM Codes of bacterial infection causes sepsis

Variable	ICD-10-CM Codes
Enterococcus infection	A4181
Streptococcal infection	A409, A408, A403, A401, A400, P361, P3619, P3610 A40
Salmonella infection	A4153
Pseudomonas infection	A4152
Escherichia coli [E. coli] infection	A4151, P364
Staphylococcus aureus infection	A410, P362, A411, A412, A4101, A4102
Anaerobes infection	P365, A414
Candidal infection	B377
Gonococcal infection	A5486
Actinomycotic infection	A427

ICD-10-CM, International Classification of Diseases 10th revision Clinical

Supplementary Table 3. ICD-9-CM and ICD-10-CM Codes of infection sites causes sepsis

Variable	ICD-9-CM Codes	ICD-10-CM Codes
Lung infection	1211	T86812, B671, T8633
Gastrointestinal infection	0080, 0081, 0084, 0083, 00809, 00804, 00801, 00800, 00803, 00802, 009, 008, 0082, 00845, 00844, 00847, 00846, 00849, 0088, 1230, 00843, 00842, 00841	A084，A088，T86852，A08，A04，A044，A049，A048
Skin tissue infection	6868, 6869, 686	L08, L0889, L088, L089
Joint bone tissue infection	73093, 7308, 7309, 73092, 73094, 73099, 73096, 73083, 73080, 73090, 73091, 73095, 73097, 73089, 73087, 73086, 73085, 73084, 73082, 73081, 73098, 730, 73088, 99666, 0985, 09859	T84619S, B672, T84619, T84619A T84629D, T84629A, T84629S ，T84629, T8603, T84619D, T86832, T8450, T8459, M01X9, M01X1, M01X0, T8450XA, T8450XD, T8450XS, T8459XS, T8459XA, M01, T8459XD, M01X, T845
Endocarditis infection	4211, 11514, 11504	I330
Intracranial infection	326	T85730, T85730S, T85730A, T85730D

ICD-9-CM, International Classification of Diseases 9th revision Clinical Modification;
ICD-10-CM, International Classification of Diseases 10th revision Clinical

Supplemental Table 4. Baseline Characteristics of Sepsis Hospitalizations With AKI From 2010-2014

Variables	2010 N	2010 %	2011 N	2011 %	2012 N	2012 %	2013 N	2013 %	2014 N	2014 %	P-value*
**N=3848**	738 2.7%		639 3.0%		721 3.3%		822 3.9%		928 4.3%		
**Age (yrs) at hospitalization**											
Median [IQR]	0 [0-9]		0 [0-12]		0 [0-12]		2 [0-12]		2 [0-12]		<0.001
<1 year	418	56.6%	357	55.9%	372	51.6%	385	46.8%	398	42.9%	<0.001
1-3	63	8.5%	38	5.9%	65	9.0%	68	8.3%	88	9.5%	
4-6	32	4.3%	32	5.0%	34	4.7%	44	5.4%	66	7.1%	
7-10	54	7.3%	43	6.7%	55	7.6%	79	9.6%	95	10.2%	
11-18	171	23.2%	169	26.4%	195	27.0%	246	29.9%	281	30.3%	
**Gender**											<0.001

Male	430	58.3%	356	55.7%	374	51.9%	475	57.8%	512	55.2%	
Female	308	41.7%	283	44.3%	347	48.1%	347	42.2%	416	44.8%	
**Race**											<0.001
White	306	41.5%	253	39.6%	281	39.0%	309	37.6%	367	39.5%	
African American	160	21.7%	129	20.2%	139	19.3%	166	20.2%	180	19.4%	
Hispanic	155	21.0%	102	16.0%	146	20.2%	166	20.2%	190	20.5%	
Asian or Pacific Islander	17	2.3%	18	2.8%	18	2.5%	31	3.8%	26	2.8%	
Native American	8	1.1%	4	0.6%	6	0.8%	6	0.7%	8	0.9%	
Other	92	12.5%	133	20.8%	131	18.2%	144	17.5%	157	16.9%	
**All-cause**	239	32.4%	200	31.3%	214	29.7%	213	25.9%	218	23.5%	<0.001

**in-hospital mortality**						
**Length of stay (days) Median [IQR]**	29 [11-74]	24 [8-67]	24 [10-63]	22 [9-52]	20 [8-52]	<0.001
**Total hospital charges Median [IQR]**	$285,409 [$100,523-$665,998]	$274,007 [$88,779-$709,322]	$322,980 [$120,541-$795,301]	$290,726 [$108,134-$761,638]	$267,581[$92,415-$746,231]	<0.001

*Significant P-values < 0.001, AKI=Acute kidney injury, IQR=interquartile range

Continual

Supplemental Table 4. Baseline Characteristics of Sepsis Hospitalizations With AKI From 2015-2019

**Variables**	**2015**		**2016**		**2017**		**2018**		**2019**		**P-value***
	**N**	**%**	**N**	**%**	**N**	**%**	**N**	**%**	**N**	**%**	

**N=7104**	1158 5.0%		1272 5.3%		1424 6.2%		1577 7.3%		1673 8.0%		
**Age (yrs) at hospitalization**											
Median [IQR]	3 [0-12]		5 [0-14]		4 [0-13]		5 [0-13]		5 [0-13]		<0.001
<1 year	464	40.1%	433	34.0%	502	35.3%	505	32.0%	513	30.7%	<0.001
1-3	133	11.5%	157	12.3%	185	13.0%	216	13.7%	243	14.5%	
4-6	73	6.3%	101	7.9%	117	8.2%	144	9.1%	147	8.8%	
7-10	116	10.0%	137	10.8%	155	10.9%	160	10.1%	174	10.4%	
11-18	372	32.1%	444	34.9%	465	32.7%	552	35.0%	596	35.6%	
**Gender**											<0.001
Male	627	54.1%	673	52.9%	755	53.0%	803	50.9%	870	52%	
Female	531	45.9%	599	47.1%	669	47.0%	774	49.1%	803	48.0%	
**Race**											<0.001
White	447	38.6%	505	39.7%	562	39.5%	694	44.0%	698	41.7%	
African American	211	18.2%	249	19.6%	286	20.1%	296	18.8%	315	18.8%	

Hispanic	260	22.5%	248	19.5%	331	23.2%	317	20.1%	374	22.4%	
Asian or Pacific Islander	42	3.6%	45	3.5%	51	3.6%	71	4.5%	58	3.5%	
Native American	8	0.7%	12	0.9%	15	1.1%	19	1.2%	22	1.3%	
Other	190	16.4%	213	16.7%	179	12.6%	180	11.4%	206	12.3%	
**All-cause in-hospital mortality**	237	20.5%	269	21.2%	248	17.4%	259	16.4%	300	17.9%	<0.001
**Length of stay (days) Median [IQR]**	20 [7-50]		15 [5-40]		13 [5-42]		14 [5-43]		12 [4-37]		<0.001
**Total hospital charges Median [IQR]**	$278,446 [$85,005-$838,207]		$202,236 [$58,977-$686,096]		$181,100 [$46,370-$658,544]		$206,998 [$55,295-$759,649]		$174,884 [$49,628-$722,867]		<0.001

*Significant P-values < 0.05, AKI=Acute kidney injury, IQR=interquartile range
